# Use Of Smartphones for Ensuring Vulnerable Road User Safety through Path Prediction and Early Warning: An In-Depth Review of Capabilities, Limitations and Their Applications in Cooperative Intelligent Transport Systems

**DOI:** 10.3390/s20040997

**Published:** 2020-02-13

**Authors:** Ioannis Vourgidis, Leandros Maglaras, Ahmed S. Alfakeeh, Ali H. Al-Bayatti, Mohamed Amine Ferrag

**Affiliations:** 1 School of Computer Science and Informatics, De Montfort University, Leicester LE1 9BH, UK; P13241213@my365.dmu.ac.uk; 2 Faculty of Computing and Information Technology, King Abdulaziz University, Jeddah 21589, Saudi Arabia; asalfakeeh@kau.edu.sa (A.S.A.); alihmohd@dmu.ac.uk (A.H.A.-B.); 3 Department of Computer Science, Guelma University, Guelma 24000, Algeria; ferrag.mohamedamine@univ-guelma.dz

**Keywords:** cooperative intelligent transport systems (C-ITS), vehicle to pedestrian (V2P), pedestrian to vehicle (P2V), vulnerable road users (VRUs), GPS, smartphones, inertial measurement units sensors

## Abstract

The field of cooperative intelligent transport systems and more specifically pedestrians to vehicles could be characterized as quite challenging, since there is a broad research area to be studied, with direct positive results to society. Pedestrians to vehicles is a type of cooperative intelligent transport system, within the group of early warning collision/safety system. In this article, we examine the research and applications carried out so far within the field of pedestrians to vehicles cooperative transport systems by leveraging the information coming from vulnerable road users’ smartphones. Moreover, an extensive literature review has been carried out in the fields of vulnerable road users outdoor localisation via smartphones and vulnerable road users next step/movement prediction, which are closely related to pedestrian to vehicle applications and research. We identify gaps that exist in these fields that could be improved/extended/enhanced or newly developed, while we address future research objectives and methodologies that could support the improvement/development of those identified gaps.

## 1. Introduction

Vulnerable road users is a collective term used to describe cyclists, motorcyclists, moped riders and pedestrians. More specifically, in one of the studies presented, the number of accidents was predicted under the acceptance that no further successful measures including Intelligent Transport Systems (ITS) solutions were to be introduced within road transport systems. For these studies, the Community Road Accident database, CARE, was used. CARE is a European Commission database resource comprising detailed data of individual accidents, from the EU member states. According to this research, during the period 2002–2012, there was a significant decline in the number of accidents overall. However, the decline in Vulnerable Road Users’ (VRU) accidents is much less recorded, with bicycle and pedestrian casualties remaining relatively constant. This research predicted that the number of bicycle accidents was expected to increase while, by 2030, the number of VRU accidents will be almost comparable to those of car accidents since there is a significant decreasing rate of those accidents [1]. Due to the fact that the number of fatalities remains high and despite the recent advances in traffic safety, the United Nations have set as one of the first priorities to halve the number of deaths and injuries from road traffic accidents by 2020 [2] while the European Commission aims at reducing the number of fatalities in road transport to nearly zero by 2050. In this context, cooperative intelligent transport systems could play a significant role, since they utilize the coordination between different traffic agents such as vehicles and vulnerable road users. In such a scenario, vehicles and VRUs could exchange information in order to prevent dangerous traffic situations. This type of connectivity is even more important via the market penetration of vehicular communication systems and the increasing use of smartphones worldwide. More specifically, smartphones could play a vital role in the development of pedestrian to vehicle communication safety systems. By 2020, 6.1 billion smartphone users are expected globally. This equals 70% of the world’s population, showing the potential of this new information source [3], while in the last quarter of 2018, the total number of mobile subscriptions was around 5.9 billion [4]. Smart devices, with their included sensors and user interface, can provide information about the current movement state (velocity, acceleration and heading) and the position and characteristics of their users, such as age, gender and habits. On the other hand, sensors installed on vehicles could also provide information about the vehicle’s current position, direction and speed but also the driver’s intentions. The concept of Cooperative Intelligent Transportation Systems (C-ITS) is to combine measurements and information from vehicles, pedestrians and local infrastructure within a limited geographical area. Current applications within this area of interest are collision avoidance systems, emergency vehicle warning systems and traffic mitigation [5].

The contributions of the paper are threefold:

- A comprehensive review of the current state-of-the-art outdoor localization methods via smartphones is conducted.
- In addition, Pedestrians to Vehicles (P2V), Systems and methods for next move/step prediction are presented and analyzed
- Finally, a Pedestrian to Vehicles framework (P2V) is proposed.

The rest of this paper is organized as follows. Section 2.1 provides an overview of related work in the domain of Cooperative Intelligent Transportation Systems (C-ITS). Section 2.2 describes several Vehicle to Pedestrians (V2P) and Pedestrians to Vehicle (P2V) systems. Section 2.3 describes VRU outdoor localization methods via smartphones. Section 3 presents VRU next move/step prediction current implementations. Section 4 provides a general overview of a prediction communication P2V proposed framework. Finally, Section 5 summarises our findings and concludes our work.

## 2. State-of-the-Art Review in Vehicle to Pedestrians Systems

### 2.1. CITS 0verview

The development of Global Navigation Satellite Systems (GNSS) along with the adoption of mobile internet allow the rapid rise of Cooperative Intelligent Transportation Systems (C-ITS). Improvement of traffic conditions and safety levels on road networks, either urban or highways, is the main goals of those systems. C-ITS technologies that are based on smartphones may contribute to real-time vehicle data collection and in traffic safety and sustainability issues. The information content is characterized by the spatial scope that could be utilized and the time period that the information remains valid [6]. Table 1, displays the local validity, the distance for which the information provided refers to, and that explicit lifetime, the time range within which information is considered to be correct, of several content types typical for applications within C-ITS [6]. The communication of information in C-ITS is performed over a vehicular ad hoc network (VANET) a type of mobile ad hoc network where vehicles are used as mobile nodes [7]. VANETs comprise both vehicle-to-vehicle (V2V) and vehicle-to-infrastructure (V2I) communications. However, despite a significant amount of conducted research, the deployment of real-world VANETs has been slow. This is due to the costs associated with the required communication devices [8]. Using smartphones in VANETs is appealing because it is cost effective while it supports the integration of pedestrians, bicyclists and motorcyclists into the network.

- Advanced Transportation Management Systems (ATMS): ATMSs aim to reduce traffic congestion instances especially in urban environments by optimizing the efficiency of usage of existing infrastructures. The optimum solutions that those systems try to find, both in urban freeways and surface streets, are based on the blending of state-of-the-art sensing, communication and data-processing technologies [9].
- Advanced Traveller Information System (ATIS): Travelers’ travel choices are mainly based on the knowledge that they gain from previous experience when traveling through areas of a city. With the use of Advanced Travel Information Systems (ATIS), which are designed to provide real-time information about available travel alternatives based on the current situation on the roads, travelers can take better travel choices. Using this technology, the experience of travelers is combined with descriptive, prescriptive and feedback information. Descriptive information consists mainly of data about prevailing conditions such as current or predicted travel times. This information can be provided either pre-trip or en-route through message signs or onboard devices. On the other hand, prescriptive information provides travelers with the “best” alternative, e.g., the route with the shortest distance, with the least total travel time or that is most eco-friendly [10,11].
- Advanced Vehicle Control and Safety System (AVCSS): These systems apply advanced technologies both in vehicles and roads to assist drivers to better control vehicles and to consequently reduce traffic accidents. The main services included in the AVCSS are longitudinal collision avoidance, lateral collision avoidance, intersection collision avoidance, vision enhancement for crash avoidance, safety readiness and pre-crash restraint deployment [12].
- Advanced Public Transportation System (APTS): Advanced public transportation systems (APTS) can be used for the improvement of both traffic efficiency of operation and the safety of public transportation users. Those systems combine transportation management and information technologies to public transit systems. Some of the most well-known APTSs are real-time passenger information systems, automatic vehicle location systems and bus arrival notification systems [9].
- Commercial Vehicle Operation (CVO): Intelligent Transport Systems (ITS) are used in commercial vehicle operations to help improve vehicle safety while at the same time can enhance the communication between motor carriers and respective regulatory agencies. Some applications of ITS are the following:
  − Safety information exchange systems: These systems can expedite the collection, distribution and retrieval of safety information.
  − Electronic screening systems: They can be used for automated inspection of vehicles.
  − Electronic credentialing systems: They can be used for electronic submission to systems, processing, approval, invoicing, payment of tolls and even tax filing [13].

**Figure 1:**
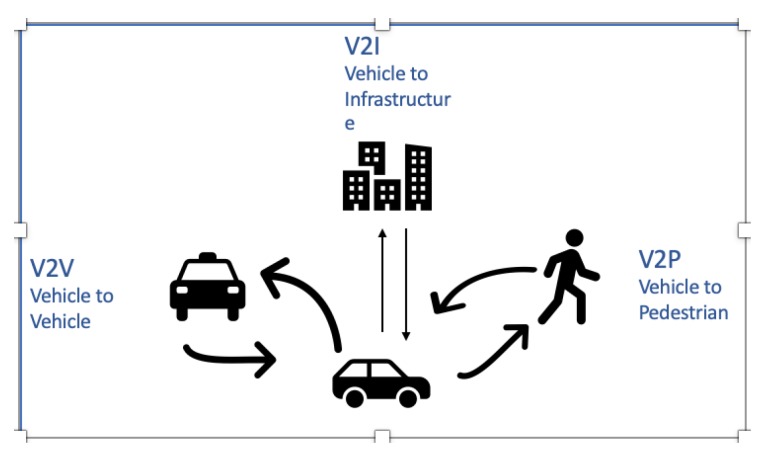
Vehicle communication types in C-ITS in Reference [2].

Figure 1 illustrates all the different vehicle communication types within C-ITS.

### 2.2. Vehicle to Pedestrians (V2P) Systems—Developments

Although pedestrians are among vulnerable road users that are involved in fatal accidents [14], previous research on road safety in VANETs is mainly focused on vehicle to vehicle accidents. Incorporating pedestrians as active players into VANETs with the use of their smartphones as communicating nodes enables the deployment of novel active road safety applications such as vehicle to pedestrian (V2P) collision avoidance. Active road safety applications are those that are primarily employed to decrease the probability of traffic accidents and the loss of life of the occupants of vehicles and vulnerable road users. Some examples of active road safety applications are intersection collision warning, emergency vehicle warning, pre-crash sensing/warning abd collision risk warning [15]

The use of mobile devices is increasing by vulnerable road users, especially pedestrians, since they rely more on mobile navigation as a means to find their way throughout the various contexts of foot travel. However, the use of mobile phones by pedestrians affects their awareness of their surrounding environment, as a result, increasing accident risk. Therefore pedestrian distraction associated with mobile phone usage is a very important contemporary safety issue, particularly relevant in urban environments with high traffic density. However, most mobile solutions neglect the risks related to the influence of mobile phone usage in high-traffic situations [16]. For that reason, safety related to the use of mobile devices is important, especially within an urban environment where traffic is of high density [17]. According to Reference [18], the introduction of autonomous vehicles is an opportunity to reduce accident rates due to human errors, since the driver’s intervention will not be required. However, the factor of trust is vital in adopting autonomization, since road users that will closely interact with autonomous vehicles will have to assess and verify autonomous vehicles’ reliability and effectiveness as far as vulnerable road user’s safety.

During the last 10 years, significant research has been conducted in the field of the C-ITS and more specifically in the field of pedestrian to vehicle and vice versa. In all of the cases, smartphone sensors were used in order to use pedestrian’s information such as position and heading to be transmitted to surrounding vehicles. As far as vehicle information, this was provided either from smartphones existing within the vehicle, already installed sensors such as GPS or, in some cases, laptops having installed required network cards, such as external WiFi, placed on the vehicle’s dashboard. Most of the authors, apart from those studying smartphone’s GPS, used data coming from the smartphone’s accelerometer, gyroscope and magnetometer. According to the results provided, GPS localization inaccuracies were pinpointed, while in the case where they tried to predict pedestrian’s next moves/short path, they pointed out the difficulties related to this process. It has to be mentioned that the majority of the authors tried to tackle non-line-of-sight and blind spots, where the driver’s view is limited due to buildings, vehicles and other obstacles. The majority of research was carried out in 2014 [14,19,20,21], in 2016 [1,22,23,24,25,26] and in 2017 [27,28,29]. Moreover, most of the V2P developments used several sensors data fusion techniques [1,19,21,22,24,26,27,30], while some of them [20,31] discussed methods to predict VRU next move/short path and some others presented collision avoidance systems [23,26,32]. Table 2 summarises in a chronological order, the most representative research carried out per year from 2008–2018 within the field of V2P/P2V.

**Figure 2:**
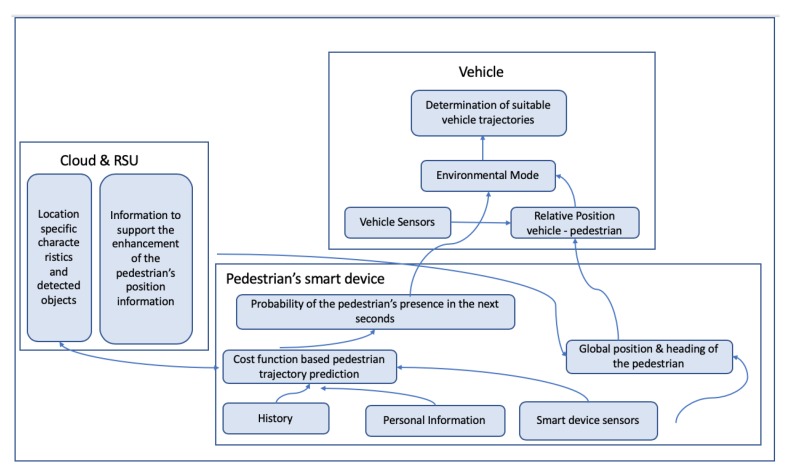
Proposed system in Reference [30].

**Figure 3:**
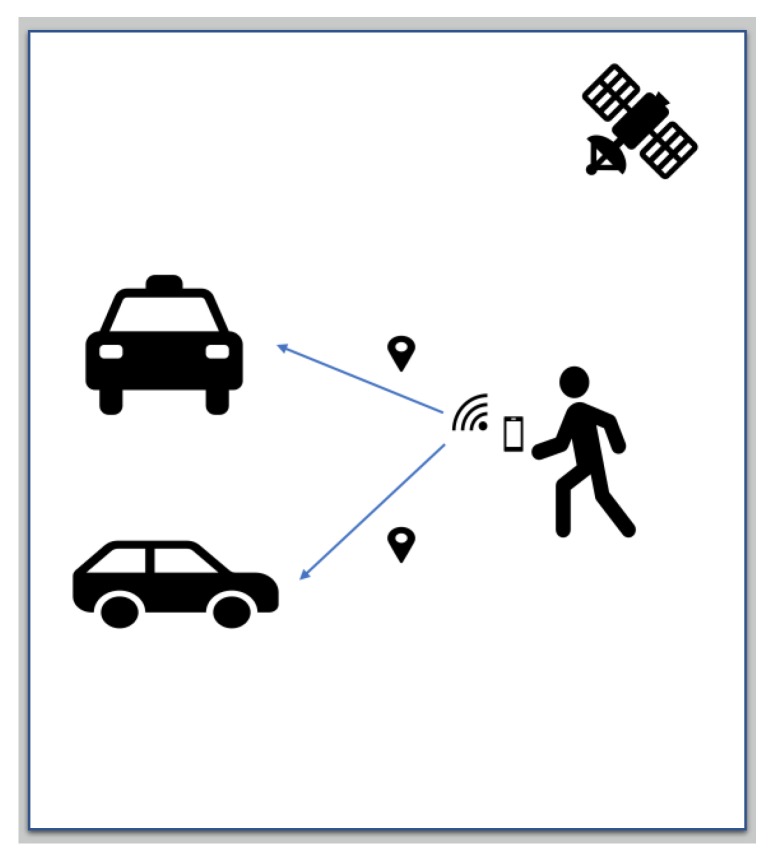
WiFi safe basic idea in Reference [35].

**Figure 4:**
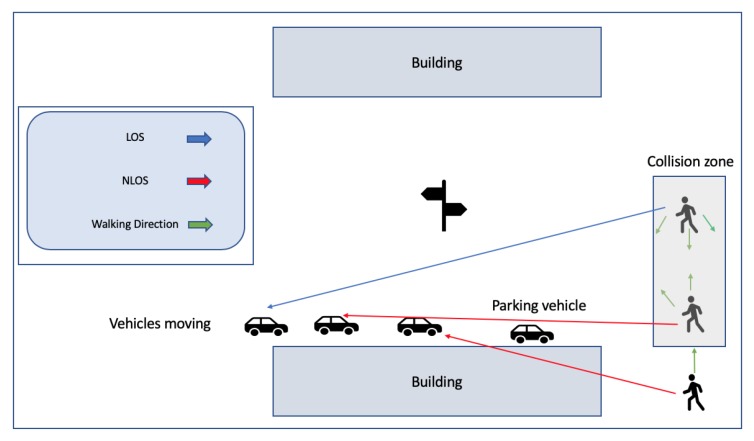
WiFi dangerous scenarios in Reference [35].

Figure 2 depicts the system design described in Reference [30], while in Figure 3 and Figure 4, the system design presented in Reference [35] is depicted.

### 2.3. VRU Outdoor Localization via Smartphones

According to the above, cooperative intelligent transport systems are about to play a significant role in traffic safety, since the objective of the United Nations is to halve the number of deaths and accidents from traffic accidents by 2020 [2]. According to the above research, within a vehicular network scenario, vehicles and vulnerable road users can exchange information such as position, speed, heading in order to prevent accidents. The increasing use of smartphones sets them as one of the most suitable candidates for serving this communication but for providing vulnerable road user’s information. Vulnerable road user’s real-time accurate position is a vital factor for the success of these systems because the more accurate real-time positioning measurements, the better information will be provided for VRU’s trajectory and heading. Within the research, there have been so far 3 position correction methods: differential GPS, smartphone sensors data fusion and multi-satellite systems.

#### 2.3.1. GPS/Assisted GPS/Differential GPS

**Figure 5:**
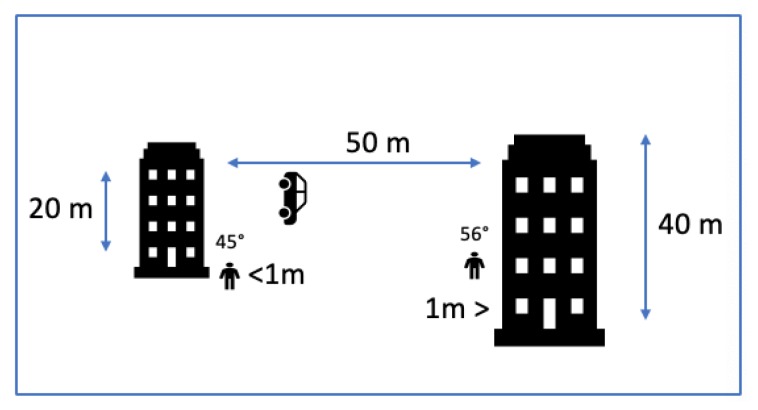
View of the sky (angle in degrees), as presented in Reference [2].

GPS is the most common global positioning technology, used to determine a three-dimensional ground location of any object. Although GPS when embedded in-vehicle navigation devices has been proved to be efficient, it has a number of shortcomings when used in pedestrian location-based services (LBS) [2]. GPS’s main drawback is low performance within “urban canyons”, places where direct lines of sight to the GPS satellites are obstructed from nearby buildings. In those situations, signals can reach the receiver after being reflected, usually many times, from nearby surfaces, known as multi-path signals [2,36]. The situation is even worse for pedestrians positioned on the pavement, close to tall buildings that obstruct the signals. Figure 5 displays the view of the sky based on proximity to buildings.

**Figure 6:**
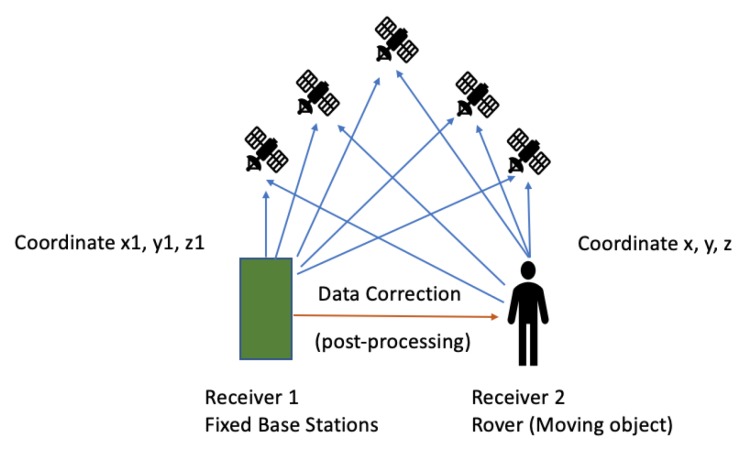
How differential GPS (DGPS) works as presented in Reference [40].

While in vehicles the GPS antennae are mounted in a place having a clear sky view, in pedestrians’ cases, smartphones are possibly in bags or pockets, resulting in signal deterioration. Moreover, GPS does not provide any ways to calculate VRU heading, which is essential for C-ITS targeting to VRU safety. Due to these reasons, additional services and sensors are required/already being used to improve VRU’s tracking [2]. Through the introduction of assisted GPS (A-GPS), the GPS receivers allow to retrieve information from network resources in order to combine them with the information received from the satellites and to thus improve location accuracy. Such systems are especially useful when the receiver is not easily reachable from satellite signals [37]. GPS-enabled smartphones are typically accurate to within a 4.9-m radius under open sky [38]. As mentioned in Reference [38], the best positional accuracy achieved was between 1 and 5 m (average error) under open sky conditions. In contrast, commercial-grade receivers with differential correction typically result in a positional error of less than 1 m [38]. Differential GPS, DGPS, is an enhancement to GPS, providing improved location accuracy from 1 to 3 m in the case of ideal implementations. DGPS combines the signals received from the satellites with those received from fixed ground-based reference stations in order to fix positioning errors. Proper digital correction signals are transmitted to all ground-based transmitters, which are called rovers. DGPS relies on two stations; one is the base station, and the next is a rover [39]. In a work developed in 2015, wheeled mobile robots were autonomously navigated and controlled. This incorporated self-localization, recovery and path planning [40]. The DGPS technique was used to develop the autonomous path following, Figure 6 illustrate how DGPS works, while the extender Kalman filter was applied to optimize the robot’s trajectory.

#### 2.3.2. Multi-Satellite Systems

In 2015, a four-system positioning model was developed by taking advantage of the available GNSS, BeiDou, Galileo, GLONASS and GPS. According to the test results, the addition of BeiDou, Galileo and GLONASS systems to the standard GPS-only processing significantly shortened the convergence time and improved the positioning accuracy [41]. These results proved that combining GPS, BeiDou, GLONASS and Galileo presents the highest accuracy. The time required to achieve the desired accuracy is several minutes for accuracy less than 10 cm, around 30 min for accuracy better than 5 cm and a few hours to reach mm-level accuracy. Therefore, it is proved that the fusion of multiple GNSS except increasing the number of observed satellites also optimizes spatial geometry parameters and thus improves convergence, accuracy, continuity and reliability of object positioning [41]. In another project carried out in 2016 [39], the authors applied a DGNSS correction project method to a commercial smartphone, while the accuracy results produced from their research are summarized in Table 3.

It was thus proved that, by integrating GLONASS into the GPS positioning system, scientists can improve root-mean-square (RMS) and mean value of errors by 18%. The RMS result for standalone GPS error has been improved to 0.42 m by applying a differential technique, and the performance of Differential GNSS (DGNSS) is better (by approximately 15%) than that of Differential GPS (DGPS).

#### 2.3.3. Inertial Navigation Systems Smartphones Sensors Data Fusion

Over the last year has been carried out significant research on the use of motion sensors, accelerometers and rotation sensors in order to calculate via the use of dead reckoning the position, the orientation and the velocity of a moving object without the aid of external systems. These sensors are usually supplemented by barometers and magnetometers. The systems that use these sensors are known as inertial navigation systems (INS). For outdoor environments, the combination of GPS and INS is commonly used to overcome the long-term drift of INS and the short outage of GPS [42]. In Refernece [43], a step-detection algorithm was introduced using one shoe-mounted accelerometer. The result of the detection algorithm was robust in several walking conditions such as walking velocity, walking type and inclination. Step length was estimated via the use of neural networks. The proposed neural network estimated the step lengths of a pedestrian well irrespective of walking frequency and inclination with an accuracy of 98% of distance traveled. In 2009, research was carried out comparing several algorithms for a pedestrian’s step detection, stride length, heading and position information by using inertial sensors, accelerometers, gyroscopes and magnetometers attached to a person’s foot [44]. The stride length results produced from the estimation algorithms, Weiberg and ZUPT [44,45] are presented in Table 4.

**Figure 7:**
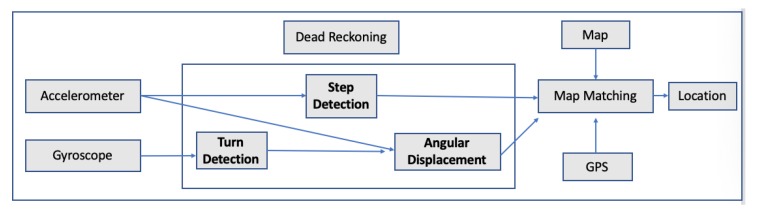
APTS’s flow chart in Reference [48].

The authors of the research implemented and tested 3 different positioning algorithms. Two of them were based on the accumulation of the estimated stride lengths, along the horizontal orientation of the foot, while the third algorithm was based on the results of the ZUPT algorithm. After a study of more than 30 test trajectories (outdoor and indoor), the error between the final position estimation and the starting point in the majority of the cases were between 5 to 15 m [43]. In 2013, another research was carried out, where GPS and low-cost INS were integrated in order to enhance pedestrian’s outdoor navigation enhanced by surrounding buildings [46]. The authors pinpointed that the ZUPT method still produces heading errors, so they developed an algorithm by using the layout of the surrounding buildings and by smoothing the errors produced by the ZUPT algorithm using extended Kalman filter. The results showed the estimated position accuracy was below 5.3 m in a 40-min walk and about 0.15% of the total distance [46]. In 2013, a system named SmartLoc was presented, estimating car location and travel distance by leveraging inertial sensors embedded in smartphones; GPS signals; and automatically detected landmarks such as bridge, traffic, lights and driving patterns such as turning, uphill and downhill from inertial sensor data to improve the localization accuracy when the GPS signal was weak. The authors tested the system within the city of Chicago and an Android smartphone mounted to the car’s windshield. The authors drove for over 100 different road segments in downtown Chicago ranging from 1 km to 10 km and over 30 km on the highway. The system’s evaluation indicates the improvement in localization accuracy compared to GPS since the error was around 20 m for 90% of travel time while the mean error of GPS was 42.2 m. The system also proved that it can also reduce energy consumption by at least 10% for localization, if GPS is periodically being turned off when its signal weak without sacrificing localization accuracy [47]. The authors in Reference [42] pinpointed that it is necessary to map am object’s position onto a spatial map in order to develop a robust navigation system. The authors of this work integrated GPS signals and data coming from INS, while they applied Kalman filter and map matching in order to identify the right trajectory and to improve the matching navigation accuracy. Kalman filter was used to fusing the data coming from the sensors, such as accelerometer and gyroscope, while the map matching algorithm was used in order to reduce the GPS positioning errors. For testing purposes, GPS receiver and MEMs2 IMU3 were mounted on a motorcycle, while the tests was performed in Tainan City. In Reference [48], the authors presented a system, named APTS, for outdoor pedestrians with smartphones. This system used a robust dead reckoning algorithm and error-tolerant algorithm for map matching. In the case, the user walks with the smartphone, the dead reckoning algorithm monitors the user’s steps and walking direction, while the user’s position is being updated according to the map matching algorithm. In case the pedestrian’s location cannot be determined due to several available routes, GPS is used to eliminate ambiguity. Hence, the main purpose of GPS is to help distinguish between distant routes; therefore, GPS is used not often. The map information stores all possible routes of the user, and when a new step or turn is being recorded by the dead reckoning algorithm, the map matching algorithm updates the pedestrian’s location. Figure 7 depicts the system’s flow.

**Figure 8:**
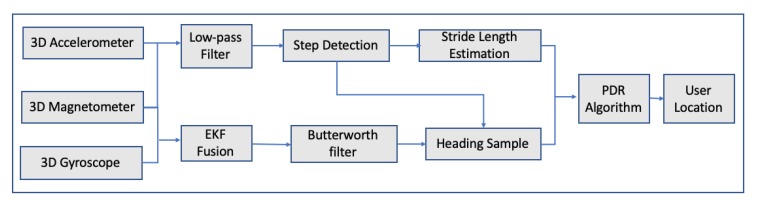
System architecture in Reference [49].

**Figure 9:**
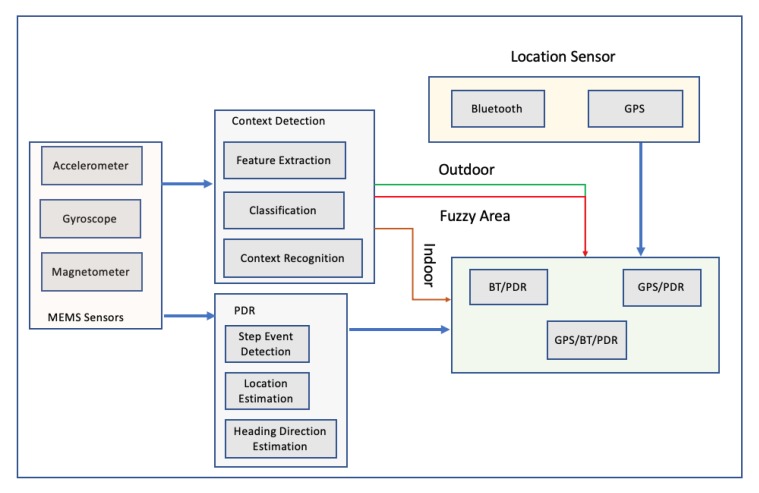
A GPS/Bluetooth/Pedestrian Dead Reckoning system design presented in Reference [50].

**Figure 10:**
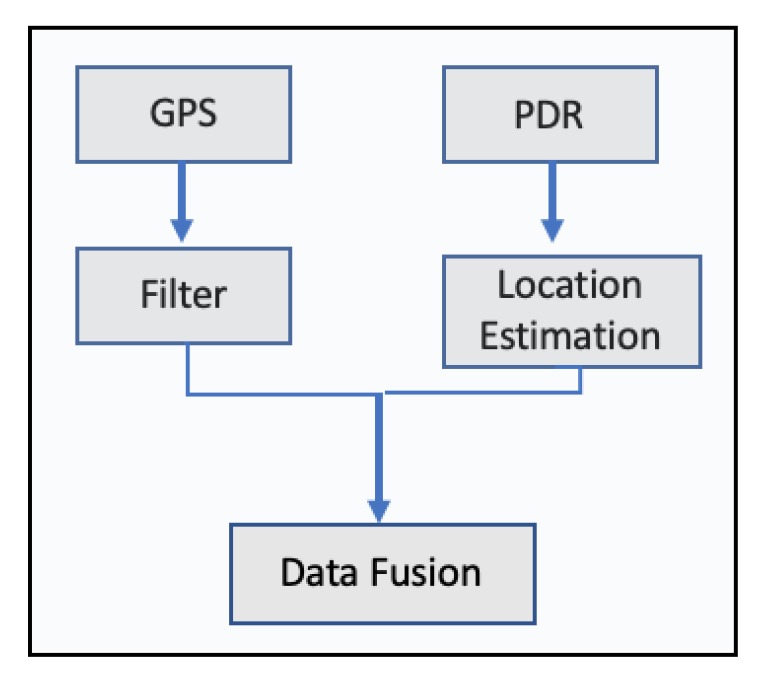
Outdoor Data Fusion presented in Reference [50].

The authors tested the system within Sunken Garden in Williamsburg, US, by using anchor points as vertices and route segments as edges, while the ground truth of the anchor points was manually found from Google Maps. The test results proved that the system produced overall fewer errors than GPS location accuracy without however performing extremely better than pure GPS. In 2014, there was another research where a pedestrian dead reckoning algorithm was introduced by using data coming from smartphone sensors [49]. The proposed algorithm consists of step detection, stride length and heading estimation. The system’s architecture is depicted in Figure 8:

For the estimation of stride length, a back propagation neural network was developed, while the extended Kalman filter was applied to determine the user’s direction. The integration of gyroscope, accelerometer and magnetometer can improve user’s heading precision while the extended Kalman filter was used to merge all the sensor’s information in order to acquire accurate information on attitude angles. During testing, a commercial smartphone and data produced from its sensors, accelerometer, magnetometer and gyroscope were collected. For the stride length estimation, data from 5 different persons with different heights were used in order to examine the performance of stride length algorithm. As far as the heading estimation test, a building’s corridors were used to assess the performance of the heading estimation algorithm, while for the trace tracking, an actual walking trace was used. The results of stride length estimation were good enough; an average standard deviation of 0.03 m for each person was achieved. As far as the heading estimation, results were not satisfactory, while the trace tracking error, on average, was 5.83 for 10 different measurements coming from 10 different people. The authors pointed out that the optimization of the heading estimation algorithm could be an interesting work in the future. In 2017, another research was published, where a smartphone-based algorithm, integrating Pedestrian Dead Reckoning (PDR)/GPS/Bluetooth for pedestrian outdoor/indoor was proposed [50]. The authors pinpointed that sensors installed in smartphones are unstable due to the interference of external magnetic, large scale iron and steel equipment. For that reason, they proposed an algorithm utilizing data from gyroscope and magnetometer to improve the accuracy of heading direction.Figure 9 illustrates the system’s design, while the data fusion process is illustrated in Figure 10.

**Figure 11:**
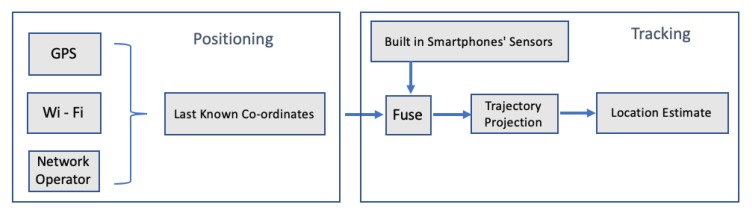
Hybrid location estimation solution as presented in Reference [51].

**Figure 12:**
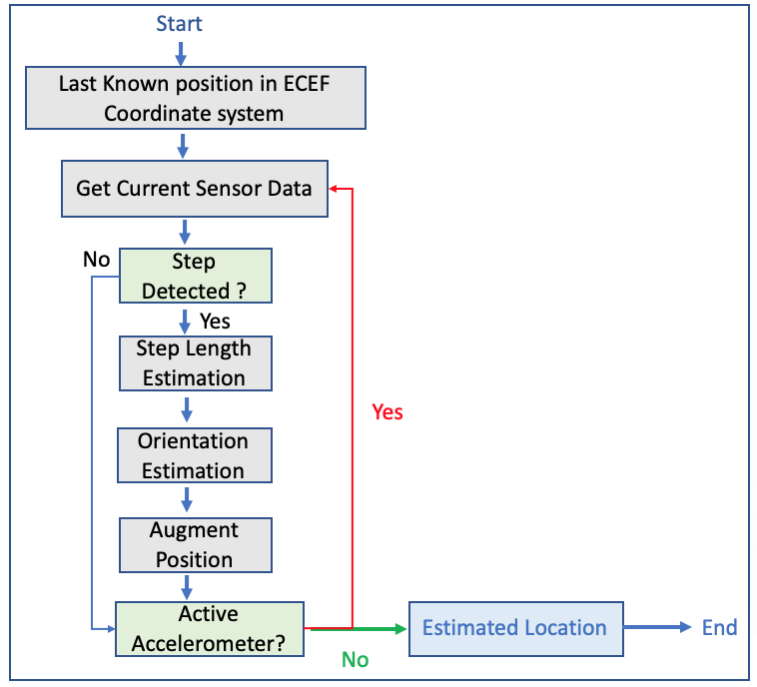
Hybrid location estimation solution as presented in Reference [51].

For the tests, the authors used a commercial smartphone and 2 people participated in the tests. During the experiment, the smartphone is held in hand and the x-axis of the phone is along the forward direction [50]. The best outdoor localization results produced where 5 m per 100 of walking distance, while the heading direction algorithm produced localization results with an error of 3 m in 70% of the test period. The authors pointed out that, in their research, they did not consider the change in height. In 2018, another research was published presenting a hybrid location estimation solution, combining GPS, pedestrian dead reckoning and a Cell-id based Trilateration algorithm (CbT) [51]. The proposed model involves two parts. The first is the static part, where the user’s position is obtained through any of the GPS, Wi-Fi or Cell-id. The second, the dynamic part, is where the user’s trajectory projection is generated via a PDR system. The Figure 11 depicts the proposed solution, while Figure 12 illustrates the proposed PDR.

The system was tested in a commercial smartphone, and the experiments were conducted within the campus of Madras Institute of Technology, Anna University, Chennai, both in indoor and outdoor environments. According to the authors, the accuracy of the Hybrid location estimation system in the case of obtaining GPS followed by PDR system is 10–15 m, while the accuracy for CbT, followed by the PDR system, is 17–25 m. The system’s execution time, running on the smartphone, was 0.14 s.

## 3. VRU’s Next Move/Step Prediction

**Figure 13:**
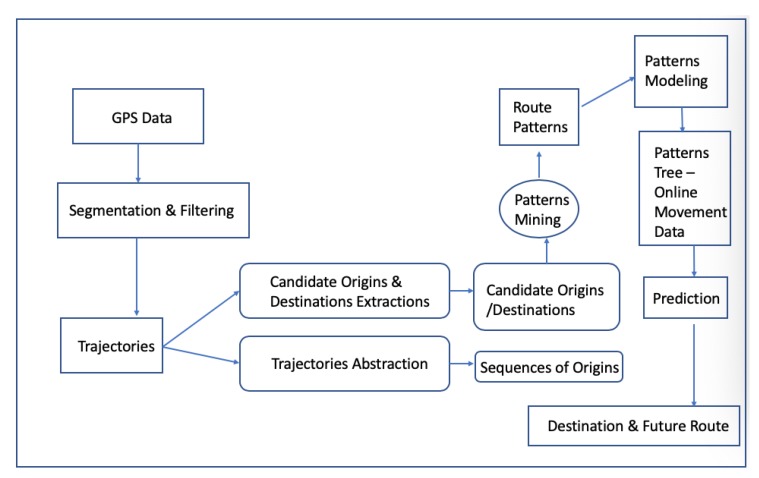
A prediction system architecture presented in Reference [52].

In Reference [52], the authors developed a system, installed on a mobile phone running on the operating system Symbian 60, to predict together the intended and the future route of a pedestrian. The authors collected GPS data from 14 participants and run several experiments to test the system’s performance. The system firstly detects the places where the person may depart from or go by using a clustering algorithm named Forward-Backward Matching (FBM). Afterward, trajectories based on a space partitioning method were abstracted and, from them, the movement patterns were extracted by applying continuous route pattern mining. These extracted movement patterns were organized in couples of origin–destination. The prediction part was made by using a decision tree created from the movement patterns. The experimental results showed that this approach achieved around 80% and 60% accuracy in destination prediction and 1-step prediction, respectively, and resulted in an average deviation of approximately 60 m in continuous future route prediction. The authors pinpointed the weaknesses of their research since they were based only on movement data. They suggested that they could use future information such as time of day, day of the week and traffic conditions. Figure 13 illustrates the prediction system’s architecture.

**Figure 14:**
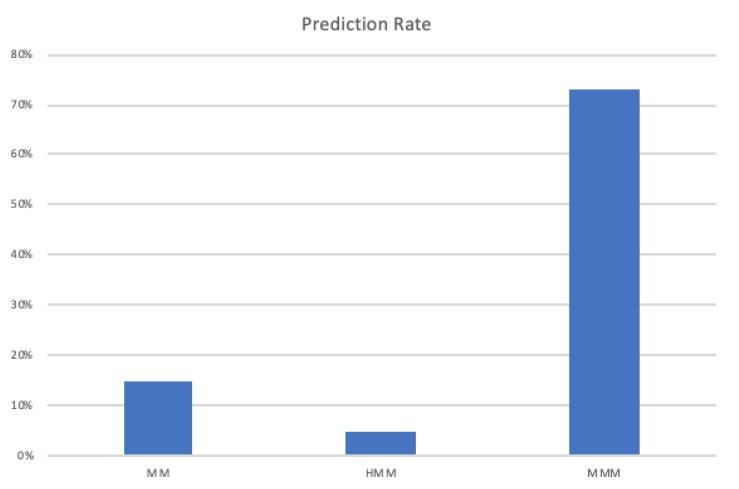
Mixed Markov–Chain Model (MMM) vs. Hidden Markon Model (HMM) vs. Markov Model (MM) step prediction rates in Reference [53].

In Reference [53], the authors presented a method to predict pedestrian’s next move by applying a mixed Markov–Chain Model (MMM) and by comparing the results against a Hidden Markon Model (HMM) and a Markov Model. The authors used 10 datasets generated by a simulator creating data from 1337 pedestrians. The prediction rates for the 3 different models applied are summarized in Figure 14.

In Reference [54], the authors applied a neural network model to predict a pedestrian’s step size within an indoor environment by using data coming from an accelerometer. The neural network was enhanced from the application of a genetic algorithm. The authors pointed out the weaknesses of their research since more sensors’ data could/should be used. In Reference [55], the authors pointed out that pedestrian path prediction is an important part of the vehicle safety system assisting in preventing possible V2P. They focused on developing a pedestrian path prediction system based on pedestrian positioning information recorded by smartphone devices. The authors developed two neural networks for this work, leveraging information such as GPS, heading, and velocity recorded by smartphones. The first neural network model was invariant to the pedestrians’ walking location and GPS noise, while the second neural network was time series neural network. The performance of those two neural networks was compared against a prediction based on dead reckoning. For the tests, 2 pedestrians participated and the walking trips were recorded (average walking trip 850 s). The results showed that the dead reckoning method makes accurate predictions when the prediction time is less than 0.5 s but makes large prediction errors when the prediction time is more than 0.5 s. The neural networks performed better in cross-training, meaning training trips from one pedestrian and test trips from a different pedestrian. The authors pointed out that future research is needed for predicting within very short period of time such as >0.5 s and <2 s. In Reference [53], the authors implemented a convolutional neural network in order to detect starting movement transitions between waiting and moving of cyclists as early as possible by using a smart device. Data of 49 cyclists were gathered during an experiment at a public intersection with bicycle lanes and walkways. The ground truth was generated from a wide-angle stereo camera and visual inspection. Their results produced an F-14 score of 97% and an average delay of 783 ms.

## 4. The Prediction and Communication P2V Proposed Framework

According to the above, so far, there has been significant research and progress in smartphone outdoor localization and communication between pedestrians and vehicles. Also, the upcoming integration of more than one GNSS, GPS, GLONASS and Galileo will further improve smartphones and consequently pedestrian’s outdoor localization. For instance, there are a couple of smartphones that use Galileo such as Phone 8 Plus, iPhone 8, iPhone 10/X, Samsung S8, S Samsung 8+, Samsung Note 8, Huawei P10 Plus, Huawei Mate 9 Pro, Huawei P10, Huawei Mate 10 Pro and Huawei Mate 9.

**Figure 15:**
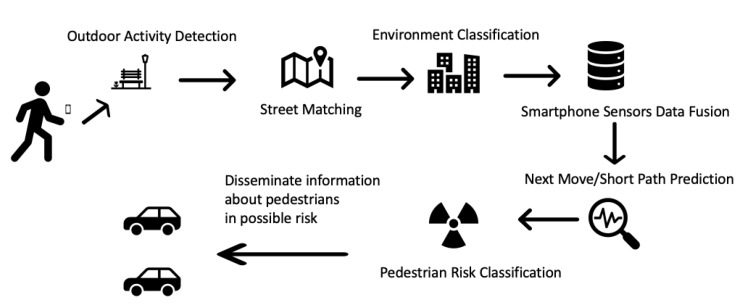
Proposed P2V framework.

However, in the field of predicting pedestrians’ next move and short path, there is room for improvement, and this is a field where this paper focuses on. This is a field, where the majority of the current research has mentioned that it should be improved in order to develop more accurate P2V systems since the localization part is being improved while efficient communication between vehicles and pedestrians has been already achieved. Based on this initial analysis, we propose a P2V framework that combines prediction and communication capabilities in order to avoid accidents between pedestrians and vehicles, especially in cases where the driver does not have visual contact with the pedestrian due to obstacles. The suggested P2V framework consists of the following stages which are illustrated in Figure 15:

- Mobile phone of each passenger collects and fuses data coming from sensors such as accelerometer, magnetometer, gyroscope, compass, GPS, WiFi, barometer if available and heart rate sensor. Also data from maps are being collected as well.
- Outdoor activity detection is carried out in order to identify whether the user is outdoor or indoor based on GPS and accelerometer data [21,56,57].
- Street Matching is being carried out in order to determine whether the user is near a street by matching his/her location against a map. Based on Reference [21], where the authors have applied map matching techniques for vehicles [58,59], a similar approach could be developed to identify on which street the pedestrian is walking.
- Environment classification is executed in order to classify the area in which the pedestrian is walking, such as rural out of town, suburban and urban [21].
- Current and historical fused data are used in order to predict the next movement/next short path of the pedestrian.
- The pedestrians are classified on whether their current and near future position would lead them to a possible risk within the next seconds (maximum 10 s).
- Only the medium-risk and high-risk classified pedestrians’ information is communicated to all neighbors such as bicycles, motorbikes and cars.
- Based on this information, all neighbouring nodes can take the appropriate decisions in order to avoid possible accidents.

Our research path for implementing this framework is the following:

- Analyse the feasibility of existing prediction models for meeting the specific requirements of our framework;
- Measure their efficiency and accuracy;
  − Test different data mining techniques such as mean absolute error, root mean squared error, mean absolute percentage error and mean absolute scaled error.The models will be evaluated in terms of the accuracy, precision and recall results;
  − Integrate those models into a simulated environment using specific simulator environments like Veins [35] where the communication and real-time reaction of different entities will be incorporated and evaluated;
  − Our main objective is for the data processing part, data fusion, outdoor activity detection, street matching, environment classification, prediction and risk classification to be executed on the pedestrians mobile phone, a matter that needs to be evaluated during simulations, in terms of performance and smartphone battery consumption.

Our research is expected to contribute to the field of real time series prediction by applying state-of-the-art methodologies in order to improve P2V safety via the use of smartphones. To the best of our knowledge, it is the first time that long short-term neural networks will be used to predict in real-time pedestrian’s next move/short path by conducting real-time analysis of data that are coming only from pedestrians’ smartphones’ sensors data.

## 5. Conclusions

According to the World Health Organization, from 2007 to 2015, approximately 1.25 million people have died each year in traffic accidents worldwide, with half of these deaths being pedestrians, cyclists or motorcyclists (VRUs). The World Health Organization has predicted that, by 2030, these traffic accidents will be the seventh leading cause of death. Cooperative intelligent transport systems and more specifically collision avoidance systems could play a vital role in mitigating this phenomenon. On the other hand, due to the increasing use of smartphones, industry and research have paid a lot of attention to the development of a specific type of collision avoidance system, P2V. It has been identified that, for the positioning accuracy provided by the integration of multiple GNSS within smartphones, the development of dead reckoning algorithms by processing data coming from smartphones’ IMS sensors and the use of proper communication techniques, P2V could play a significant role in minimizing these kind accidents. However, according to the literature, there is a room for improvement in predicting VRU’s next move/next short path. The current article proposes a prediction communication P2V framework that can provide enhanced road safety to VRUs. 

## Figures and Tables

**Table 1:** Cooperative Intelligent Transport Systems (C-ITS) information lifetime and local validity in Reference [6].

Explicit Lifetime	Local Validity (km)
0–30 s Accident Warning	0.1
31 s–10 min Emergency Vehicle Warning	1
1 day–work zone warning	5

**Table 2:** P2V/V2P literature review summary.

Reference	Year	Sensors	Method	System Type
[33]	2008	GPS	Collision Risk Evaluation	Pedestrian-to-vehicle communication system
[32]	2011	GPS, Accelerometer	Pedestrian Movement Recognition	Collision Avoidance
[34]	2011	Accelerometer	Prediction of Pedestrian Behavior	Patented Method for avoiding collision
[31]	2013	GPS, Accelerometer, Gyroscope, Compass	Dead Reckoning Algorithm	GPS Positioning in VRU Protection Systems
[14]	2014	GPS	V2P Wireless Communication	Cellular technologies user for for V2P applications
[19]	2014	Accelerometer, Gyroscope, Compass	Sensors Fusion	Driver Detection System
[20]	2014	Wi-Fi, GPS, Gyroscope, Accelerometer, Magnetometer	Pedestrian/Vehicle Path Prediction	A DSRC based vehicle-pedestrian safety system
[21]	2014	Accelerometer, GPS	Smartphone Sensors Fusion	Pedestrians risk classification
[30]	2015	GPS, Accelerometer, Gyroscope, Compass, Gravity, Magnetometer	Smartphone Sensors Fusion	Sensing unsafe pedestrian movements
[1]	2016	GPS, Accelerometer, Gyroscope, Compass, Gravity, Magnetometer	Sensors Fusion	Smartphone Based Transport Safety System
[22]	2016	GPS, Accelerometer, Gyroscope	Sensors Fusion	Pedestrian Safety with mobile crowd sensing
[23]	2016	GPS, Magnetometer	Collision Prediction Algorithm	Collision Prediction Algorithm for P2V and V2P
[24]	2016	GPS, Accelerometer, Gyroscope, Magnetometer	Sensors Fusion	Traffic safety framework by sensing driving behavior
[25]	2016	GPS	Vehicle GPS Data Fusion	V2P to enhance VRUs’ safety
[26]	2016	GPS, Acccelerometer, Magnetometer, Gyroscope	Sensors Fusion, Collision Prediction	VRU protection system
[27]	2017	GPS, Acccelerometer	Sensors Fusion, VRU Context/Activity	Smartphone collision avoidance system
[28]	2017	GPS, Accelerometer, Gyroscope, Magnetometer	VRUs Future Position Prediction	V2X pedestrian collision avoidance system
[29]	2017	GPS, WiFi	VRUs position broadcast via WiFi	Wi-Fi Pedestrian Collision Avoidance System

**Table 3:** Analysis results of position accuracy for GPS, multi-Global Navigation Satellite System (GNSS) and differential methods in Reference [39].

Assessment Method	GPS–DGPS	Glonass–DGPS	Multi GNSS–DGNSS
RMS (m)	0.42	0.64	0.41
Mean (m)	0.46	0.44	0.30

**Table 4:** Performance of two stride length estimation algorithms for 3 different walking speeds [24,25].

SL Algorithm	Walking Algorithm	Total Distance Error	Total Travel Distance % Error
Weiberg	Slow	−1.10 m	0.30%
	Normal	−2.64 m	0.73%
	Fast	2.18 m	0.78%
ZUPT	Slow	−2.23 m	0.62%
	Normal	4.15 m	1.15%
	Fast	−3.47 m	0.97%
